# Must Fault Localization for Program Repair

**DOI:** 10.1007/978-3-030-53291-8_33

**Published:** 2020-06-16

**Authors:** Bat-Chen Rothenberg, Orna Grumberg

**Affiliations:** 8 Microsoft Research Lab, Redmond, WA USA; 9 University of Southern California, Los Angeles, CA USA; Technion - Israel Institute of Technology, Haifa, Israel

## Abstract

This work is concerned with fault localization for automated program repair.

We define a novel concept of a *must* location set. Intuitively, such a set includes at least one program location from every repair for a bug. Thus, it is impossible to fix the bug without changing at least one location from this set. A fault localization technique is considered a *must* algorithm if it returns a must location set for every buggy program and every bug in the program. We show that some traditional fault localization techniques are not must.

We observe that the notion of must fault localization depends on the chosen repair scheme, which identifies the changes that can be applied to program statements as part of a repair. We develop a new algorithm for fault localization and prove that it is *must* with respect to commonly used schemes in automated program repair.

We incorporate the new fault localization technique into an existing mutation-based program repair algorithm. We exploit it in order to prune the search space when a buggy mutated program has been generated. Our experiments show that must fault localization is able to significantly speed-up the repair process, without losing any of the potential repairs.

## Introduction

Fault localization and automated program repair have long been combined. Traditionally, given a buggy program, fault localization suggests locations in the program that might be the cause of the bug. Repair then attempts to change those suspicious locations in order to eliminate the bug.

Bad fault localization may cause a miss of potential repairs, if it is too restrictive, or cause an extra work, if it is too permissive. Studies have shown that for test-based repair imprecise fault localizations happen very often in practice [27]. This identifies the need for fault localization that can narrow down the space of candidates while still promising not to lose potential causes for a bug.

In this work, we define the concept of a *must* location set. Intuitively, such a set includes at least one location from every repair for the bug. Thus, it *must* be used for repair. In other words, **it is impossible to fix the bug using only locations outside this set**. A fault localization technique is considered a *must* algorithm if it returns a must location set for every buggy program and every bug in the program.

**Fig. 1.**
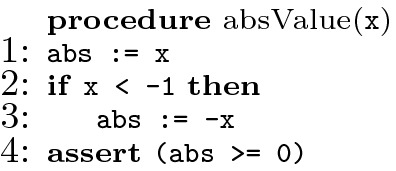
A buggy program

To demonstrate the importance of the *must* notion, consider the program in Fig. 1 for computing the absolute value of a variable x. The program is buggy since the assertion in location 4 is violated when initially $\texttt {x = -1}$. Intuitively, a good repair would replace the condition $\texttt {(x < -1)}$ in location 2 with condition $\texttt {x <= -1}$. Our must fault localization, defined formally in the paper, will include location 2 in the must location set. In contrast, the fault localization techniques defined for instance in [14, 21] do not include 2 in their location sets: They are not must and may miss optional repairs.

Our first observation regarding must notions is that their definition should take into account the *repair scheme* under consideration. A repair scheme identifies the changes that can be applied to program statements as part of a repair. A scheme can allow, for instance, certain syntactic changes in a condition (e.g. replacing $\texttt {<}$ with $\texttt {>}$) or in the right-hand-side expression of an assignment (e.g. replacing + by -). A particular location set can be a *must* set using one scheme, but non-*must* using another. We further discuss this observation when presenting our formal definition of a must fault localization.

The setting of our work is as follows. Our approach is formula-based rather than test-based. We handle simple C-programs, with specification given as assertions in the code. Similarly to bounded model checking tools (e.g. [8]), the program and the negated specification are translated to a set of constraints, whose conjunction forms the *program formula*. This formula is satisfiable if and only if the program violates an assertion, in which case a satisfying assignment (also called a *model*) is returned.

We focus on a simple repair scheme of syntactic changes, as described above. We assume that the user prefers repairs that are as close to the original program as possible and will want to get several repair suggestions. Thus, we return *all* *minimal repairs* (minimal in the number of changes applied to the program code).

Once the notion of must fault localization is defined, we develop a new algorithm for fault localization and prove that it is *must* with respect to syntactic mutation schemes. The input to the algorithm is a program formula $\varphi$ and a model $\mu$ for $\varphi$, representing a buggy execution of the program. Our approach is based on a dynamic-slicing-like algorithm that computes dependencies.

For a variable *v* in $\varphi$, its slice *F* is computed based on dynamic dependencies among variables in $\varphi$, whose values influence the value of *v* in $\mu$. Informally, *F* is a must location set that contains all assignment to the variables that *v* depends upon. Some assignment from *F* thus must be changed in order to eliminate the bug associated with $\mu$.

We incorporated the new fault localization technique into an existing mutation-based program repair algorithm [38]. In [38], the repair scheme is based on a predefined set of mutations. Given a buggy program *P*, the goal of the algorithm is to return all minimal repairs for *P*. The algorithm goes through iterations of generate-validate, where the generate part produces a mutated program of *P* and the validate part checks whether it is bounded-correct. The bottleneck of the algorithm is the size of the search space, consisting of all possible mutated programs of *P*. In [38], the search space has been pruned when the generated mutated program has been successfully validated. No pruning has been applied otherwise.

In this work, we exploit our novel *must* fault localization in order to prune the search space when a buggy mutated program $P'$ has been generated (i.e. validation failed). In this case, we compute the *must* location set *F* of $P'$. We can now prune from the search space any mutated program whose *F* locations are identical to those of $P'$. This is because, by the property of *must* location set, it is guaranteed that the bug cannot be repaired without changing a location in *F*. Thus, a large set of buggy mutated programs is pruned, without the need for additional validation and without losing any minimally repaired program. It should be noted that the smaller *F* is, the larger the pruned set is. Our experimental results confirm the effectiveness of this pruning by showing significant speedups.

To summarize, the contributions of this work are:

1. We define a novel notion of *must* fault localization with respect to a repair scheme. We show that many of the formula-based techniques are not must.
2. We present a novel fault localization technique and prove that it is *must* for the scheme of syntactic mutations. Our technique also has other advantages, such as low-complexity and incrementality.
3. We show how our new fault localization technique can be incorporated into an existing mutation-based program repair algorithm for pruning its search space. The technique is applied iteratively, whenever a generated mutated program is found to be incorrect.
4. We implemented the algorithm of repair with fault localization as part of the open source tool AllRepair. Our experimental results show that fault-localization is able to significantly speed-up the repair process, without losing any of the potential repairs.

## Motivating Example

Figure 1 presents a simple program for computing the absolute value of a variable x. The result is computed in the variable abs, and the specification states, using an assertion on line 4, that in the end abs should always be non-negative. Unfortunately, the program has a bug. The true branch of the if is intended to flip the sign of x whenever x is negative, but it accidentally misses the case where x is $-1$. As a result, if x is $-1$, the wrong branch of the if is taken, and the assertion is reached with $\texttt {abs}=-1$, which causes a violation.

Clearly, it is desirable that line number 2 be returned when running fault localization on this bug, as a human written repair is likely to change the condition on this line from $\mathtt{x < -1}$ to $\texttt {x <= -1}$ or $\texttt {x < 0}$. But, as we will show next, some of the existing formula-based fault localization techniques do not include this line in their result.

The error trace representing the bug for input $I=\{\texttt {x}\leftarrow -1\}$ is $\pi =<1,2,4>$ (this is the sequence of program locations visited when executing the program on $I$). The MAX-SAT-based fault localization technique of [21] and the error-invariant-based technique of [14] use a formula called the *extended trace formula* in order to find faulty statements along the error trace. The extended trace formula for the bug in question is

$$\underbrace{(x=-1)}_\text {Input}\ \wedge \ \underbrace{(abs=x)\ \wedge \ (x\ge -1)}_\text {Computation}\ \wedge \ \underbrace{(abs\ge 0)}_\text {Assertion}$$

This formula encodes three things: a) that the input remains $I$, b) that the computation is as the trace dictates, and, c) that the assertion holds at the end. Therefore, the formula is unsatisfiable. Both [21] and [14] intuitively look for explanations of its unsatisfiability, and therefore decide that the statement $(x\ge -1)$ on line 2 is irrelevant; The formula remains unsatisfiable even if the constraint $(x\ge -1)$ is removed.

Even the method of [6], which suggests a flow-sensitive encoding of the extended trace formula, with the goal of including all statements affecting control-flow decisions that are relevant to the bug, classifies the statement on line 2 as irrelevant. This is because the error trace does not include any location from the body of the branch that was taken (in our case it is the else branch, which is empty), in which case the flow-sensitive formula remains identical to the traditional formula.

The dynamic slicing method of [2, 23] also fails to include line 2 in its result. This method computes the set of statements influencing the evaluation of the assertion along the trace, using data and control dependency relations. A statement $st_1$ is data dependent on $st_2$ iff $st_1$ uses a variable $x$, and $st_2$ is the last to assign a value to $x$ along the trace. In our example, the assertion on line 4 is data dependent only on the statement in line 1, which in itself is not data dependent on any other statement. A statement $st_1$ is control dependent on a conditional statement $st_2$ iff $st_1$ is inside the body of either branch of $st_2$. None of the statements along our error trace is control dependent on another statement. The slice, which is the set of lines returned, is computed using the transitive closure of these relations. Thus, for our example, only line 1 is part of the slice.

In this example, we have seen how many different fault localization techniques fail to include a statement that is relevant, i.e., where a modification could be made for the bug to be fixed. In contrast, the set of locations returned by our technique for this example is $\{1,2\}$. The fact that our technique includes line 2 is not a coincidence: We show that, intuitively, whenever a repair can be made by making changes to a single line, this line *must* be included in the result. In general, whenever a repair can be made by making changes to a set of lines, at least one of them must be included in the result.

**Fig. 2.**
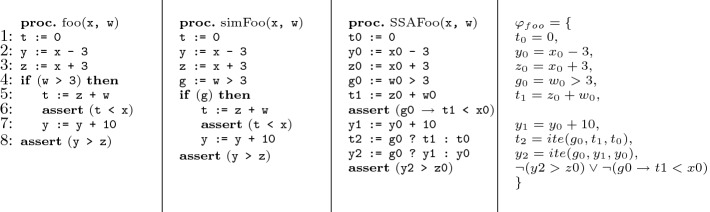
Example of the translation process of a simple program

## Preliminaries

### Programs and Error Traces

For our purposes, a *program* is a sequential program composed of standard statements: assignments, conditionals, loops and function calls, all with their standard semantics. Each statement is located at a certain *location* (or *line*) ${l}_i$, and all statements are defined over the set of program variables $X$.

In addition to the standard statements, a program may also contain *assume* statements of the form assume(bexpr), and *assert* statements of the form assert(bexpr). In both cases bexpr is a boolean expression over $X$. If an assume or an assert statement is located in ${l}_i$, execution of the program stops whenever location ${l}_i$ is reached in a state where bexpr is evaluated to false. In the case of an assertion, this early termination has the special name *assertion violation*, and it is an indication that an error has occurred.

A program $P$ has a *bug on input *$I$ if an assertion violation occurs during the execution of $P$ on $I$. Otherwise, the program is *correct for *$I$.^1^ Whenever $P$ has a bug on $I$, this bug is associated with an *error trace*, which is the sequence of statements visited during the execution of $P$ on $I$.

### From Programs to Program Formulas

In this section we explain how a program is translated into a set of constraints, whose conjunction constitutes the program formula. In addition to constraints representing assignments and conditionals, such a formula includes constraints representing assumptions and a constraint representing the negated conjunction of all assertions. Thus, a satisfying assignment (a *model*) of the program formula represents an execution of the program that satisfies all assumption but violates at least one assertion. Such an execution is a *counterexample*.

The translation, following [8], goes through four stages. We refer to the example in Fig. 2 to demonstrate certain steps.

1. Simplification: Complex constructs of the language are replaced with equivalent simpler ones. Also, branch conditions are replaced with fresh boolean variables. In the example, the if condition $\texttt {(w > 3)}$ is assigned to a fresh boolean variable g. Branching is then done based on the value of g, instead of $\texttt {(w > 3)}$.
2. Unwinding: The body of each loop and each function is inlined ${wb}$ times. The set of executions of the new program is called the ${wb}$-executions of $P$.
3. Conversion to SSA: The program is converted to static single assignment (SSA) form, which means that each variable in the new program is assigned at most once. This is done by replacing all variables with indexed variables, and increasing the index of a variable whenever it appears on the left-hand-side of an assignment. In the example, the first assignment to t is replaced by an assignment to t0 and the second, by an assignment to t1. Since t is assigned inside a conditional statement and is used after the statement, the if-then-else assignment t2 := g0?t1:t0 is inserted in order to determine which copy of t should be used after the conditional statement. These special if-then-else assignments are called $\varPhi$*-assignments*. In the example, there is also a $\varPhi$-assignment for y (y2=g0?y1:y0). Note that, assertions are also expressed by means of indexed variables. The specific indices in the assertion indicate the location in the execution in which the assertion is checked. In addition, if an assumption or an assertion is located within an if statement with branch condition *g*, then it is implied by *g* if it is within the then part of the if and is implied by $\lnot g$, if it is within the else part. In the example, $\texttt {assert (t < x)}$ is encoded by $(g_0 \rightarrow t_1 < x_0)$.
4. Conversion to SMT constraints: Once the program is in SSA form, conversion to SMT is straightforward: An assignment x:=e is converted to the constraint $x=e$; A $\varPhi$-assignment x:= b?x1:x2 is converted to the constraint $(x=ite(b,x_1,x_2))$, which is an abbreviation of $((b\wedge x=x_1)\vee ( \lnot b\wedge x=x_2))$; An assume statement assume(bexpr) is converted to the constraint $\texttt {bexpr}$, and an assert statement assert(bexpr) is converted to the constraint $\lnot$ bexpr (since a model of the SMT formula should correspond to an assertion violation).
  If the program includes several assertions, then they are converted to one constraint, representing the negation of their conjunction. In the example, the two assertions are converted to the following constraint:
  $$\lnot ({y2 > z0})\vee \lnot (g0 \rightarrow t1 < x0).$$
  We say that a constraint *encodes* the statement it came from and we partition constraints into three sets, $S_{assign},S_{phi}$ and $S_{demand}$, based on what they encode. $S_{assign}$ contains constraints encoding assignments, including those originated from assigning a fresh boolean variable with a branching condition; $S_{phi}$ - encoding $\varPhi$-assignments; and $S_{demand}$ - encoding demands from assert and assume statements. In particular, it encodes the negated conjunction of all assertions.

The triple $(S_{assign},S_{phi},S_{demand})$ is called a *program constraint set*. The program constraint set we get from a program $P$ when using $wb$ as an unwinding bound is denoted ${CS^{wb}_{P}}$. The *program formula* ${\varphi ^{wb}_{P}}$, is the conjunction of all constraints in all three sets of ${CS^{wb}_{P}}$:

$${\varphi ^{wb}_{P}} = (\bigwedge _{s\in S_{assign}}s)\wedge (\bigwedge _{s\in S_{phi}}s)\wedge (\bigwedge _{s\in S_{demand}}s).$$

#### Theorem 1

**(**[9]**).** A program ${P}$ is $wb$-violation free iff the formula ${\varphi ^{wb}_{P}}$ is unsatisfiable.

For simplicity of notation, in the rest of the paper we omit the superscript $wb$.

Since the program formula is the result of translating an SSA program, the formula is defined over indexed variables. Further, each constraint in $S_{assign}$ corresponds to the single variable, which is assigned in the statement encoded by the constraint.

## Must Fault Localization

In this section, we precisely define when a location should be considered relevant for a bug. This definition is motivated by a repair perspective, taking into account which changes can be made to statements in order to repair a bug.

In order to define the changes allowed, we use repair schemes. A *repair scheme* $\mathcal {S}$ is a function from statements to sets of statements. An $\mathcal {S}$*-patch* for a program $P$ is a set of pairs of location and statement $\{(l_1,st_1^r),\cdots ,(l_k,st_k^r)\}$, for which the following holds: for all $1\le i\le k$, let $st_i$ be the statement in location $l_i$ in $P$, then $st_i^r\in {\mathcal {S}}({st_i})$. The patch is said to be *defined over* the set of locations $\{l_1,\cdots ,l_k\}$. Applying an $\mathcal {S}$*-patch* $\tau$ to a program $P$ means replacing for every location $l_i$ in $\tau$, the statement $st_i$ with $st_i^r$. This results in an $\mathcal {S}$*-patched* program of $P$. The set of all $\mathcal {S}$*-patched* programs created from a program $P$ is the $\mathcal {S}$*-search space* of $P$.

Let $P$ be a program with a bug on input $I$, and $\mathcal {S}$ be a repair scheme. An $\mathcal {S}$*-repair* for $I$ is an $\mathcal {S}$-patched program that is correct for $I$. An $\mathcal {S}$*-repairable set* is a set of locations $F$ such that there exists an $\mathcal {S}$-repair defined over $F$. An $\mathcal {S}$-repairable set is *minimal* if removing any location from it makes it no longer an $\mathcal {S}$-repairable set. A location is $\mathcal {S}$*-relevant* if it is a part of a minimal $\mathcal {S}$-repairable set.^2^

In this paper, we focus on two repair schemes that are frequently used for automated program repair: the arbitrary scheme ($\mathcal {S}_{arb}$) and the mutation scheme ($\mathcal {S}_{mut}$). Both schemes only manipulate program expressions, but the mutation scheme is more restrictive than the arbitrary scheme: ${\mathcal {S}_{arb}}({st})$ is the set of all options to replace the expression of $st$^3^ with an arbitrary expression, while ${\mathcal {S}_{mut}}({st})$ only contains statements where the expression in $st$ is mutated according to a set of simple syntactic rules. The rules we consider are replacing a + operator with a - operator, and vice versa, replacing a $\texttt {<}$ operator with a $\texttt {>}$ operator, and vice versa, and increasing or decreasing a numerical constant by 1.^4^

### Example 1

In this example we demonstrate how different repair schemes define different sets of relevant locations. Consider again the foo program from Fig. 2. This program has a bug on input $I=\texttt {x}\leftarrow 0,\texttt {w}\leftarrow 0$. The error trace associated with the bug is $\langle 1,2,3,4,8 \rangle$ (the assertion on line 8 is violated).

The location set $\{3,4\}$ is a minimal $\mathcal {S}_{mut}$-repairable set: It is an $\mathcal {S}_{mut}$-repairable set because applying the $\mathcal {S}_{mut}$-patch ${\{(3,\texttt {z:=x-3}),(4,\texttt {w<3})\}}$, results in an $\mathcal {S}_{mut}$-patched program that is correct for $I$. This set is also minimal, because none of the $\mathcal {S}_{mut}$-patches defined over $\{3\}$ or $\{4\}$ alone is an $\mathcal {S}_{mut}$-repair for $I$: Each one of the $\mathcal {S}_{mut}$-patches ,  results in an assertion violation for $I$.

On the other hand, $\{3,4\}$ is *not* a minimal $\mathcal {S}_{arb}$-repairable set: For example, the $\mathcal {S}_{arb}$-patch  is an $\mathcal {S}_{arb}$-repair for $I$. Note that, the $\mathcal {S}_{arb}$-patch only needs to repair the bug, and not the program. That is, it is sufficient that there is no assertion violation on the specific input $I$, even though an assertion could be violated in the $\mathcal {S}_{arb}$-patched program on another input.

The set of all minimal $\mathcal {S}_{arb}$*-repairable set*s is $\{\{2\},\{3\},\{4,5\}\}$. Therefore, the set of $\mathcal {S}_{arb}$-relevant statements is $\{2,3,4,5\}$. The set of all minimal $\mathcal {S}_{mut}$*-repairable set*s is $\{\{2,3\},\{3,4\}\}$. Therefore, the set of $\mathcal {S}_{mut}$-relevant statements is $\{2,3,4\}$.

Fault localization should focus the programmer’s attention on locations that are relevant for the bug. But, returning the exact set of $\mathcal {S}$*-relevant* locations, as defined above, can be computationally hard. In practice, what many fault localization algorithms return is a set of locations that *may* be relevant: The returned locations have a higher chance of being $\mathcal {S}$-relevant than those who are not, but there is no guarantee that all returned locations are $\mathcal {S}$-relevant, nor that all $\mathcal {S}$-relevant locations are returned. We call such an algorithm *may fault localization*. In contrast, we define *must fault localization*, as follows:

### Definition 1

**(**$\mathcal {S}$**-must location set).** An $\mathcal {S}$*-must location set* is a set of locations that contains at least one location from each minimal $\mathcal {S}$-repairable set.^5^

### Definition 2

**(**$\mathcal {S}$**-must fault localization).** An $\mathcal {S}$*-must fault localization* algorithm is an algorithm that for every program $P$ and every buggy input $I$, returns an $\mathcal {S}$-must location set.

Note that, an $\mathcal {S}$-must location set is not required to contain all $\mathcal {S}$-relevant locations, but only one location from each minimal $\mathcal {S}$-repairable set. Still, this is a powerful notion since it guarantees that no repair is possible without including at least one element from the set.

Also note, that the set of all locations visited by $P$ during its execution on $I$ is always an $\mathcal {S}$-must location set. This is because any $\mathcal {S}$-patch where none of these locations is included is definitely **not** an $\mathcal {S}$-repair, since the same assertion will be violated along the same path. However, this set of locations may not be minimal. In the sequel, we aim at finding small $\mathcal {S}$-must location sets.

### Example 2

Continuing the previous example, the set $\{2,3,4\}$ is an $\mathcal {S}_{arb}$-must location set, and also an $\mathcal {S}_{mut}$-must location set. In contrast, the set $\{2,3\}$ is only an $\mathcal {S}_{mut}$-must location set, but not an $\mathcal {S}_{arb}$-must location set, since it does not contain any location from the $\mathcal {S}_{arb}$-minimal repairable set $\{4,5\}$. The set $\{2\}$ is neither an $\mathcal {S}_{arb}$-must location set nor an $\mathcal {S}_{mut}$-must location set.

### Example 3

Consider again the absValue procedure of Fig. 1. The set $\{2\}$ is an $\mathcal {S}_{mut}$-minimal repairable set and an $\mathcal {S}_{arb}$-minimal repairable set for the bug in question. Therefore, we can say that all algorithms that were shown in Sect. 2 not to include the location 2 in their result [2, 6, 14, 21, 23], are neither $\mathcal {S}_{arb}$-must nor $\mathcal {S}_{mut}$-must fault localization algorithms.

## Fault Localization Using Program Formula Slicing

In this section we formally define the notion of slicing. Based on this, we present an algorithm for computing must fault localization for $\mathcal {S}_{arb}$ and $\mathcal {S}_{mut}$.

### Program Formula Slicing

A central building block in our fault localization technique is *slicing*. But, we do not define slicing in terms of the program directly, but in terms of the program formula representing it, instead. The input to the slicing algorithm is a program formula $\varphi _{}$, a model $\mu$ of it, and a variable $v$. Recall that $\varphi _{}$ is a conjunction of constraints from $S_{assign},S_{phi}$ and $S_{demand}$ (see Sect. 3.2). The goal of the slicing algorithm is to compute the *slice* of the variable $v$ with respect to $\varphi _{}$ and $\mu$. Intuitively, this slice includes the set of all constraints that influence the value $v$ gets in $\mu$.

Similar to traditional slicing, it is easy to define the slice as the reflexive-transitive closure of a dependency relation. But, unlike traditional slicing, which defines dependencies between statements, our dependency relation is between variables of the formula. These variables are indexed. Each originates from a variable of the underlying SSA program, where it was assigned at most once. We refer to variables never assigned as *input variables*, and denote the set containing them by $InputVars$. A variable $v$ that was assigned once is called a *computed variable*, and the (unique) constraint encoding the assignment to it is denoted $Assign(v)$. The set of all computed variables is denoted $ComputedVars$. We also denote by $vars({e})$ the set of variables that appear in a formula or expression *e*.

**Fig. 3.**
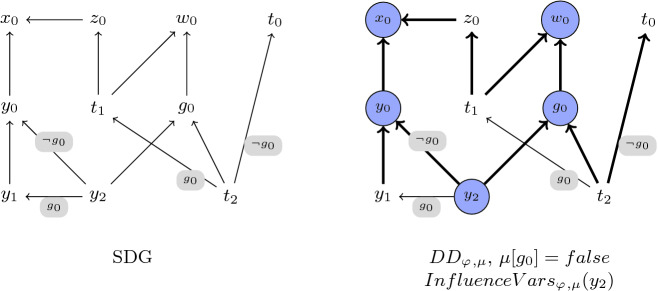
Illustration of the static and dynamic dependency relations of the foo procedure

#### Definition 3

**(Static Dependency).** The static dependency relation of a program formula $\varphi _{}$ is $SD_{\varphi _{}}\subseteq vars({\varphi _{}})\times vars({\varphi _{}})$ s.t.

$$SD_{\varphi _{}}=\{(v_1,v_2)\ |\ \exists e\ s{.}t.\ (v_1=e)\in S_{assign},v_2\in vars({e})\}\cup $$

$$\{(v, b), (v,v_1), (v,v_2)|\ (v=ite(b,v_1,v_2))\in S_{phi}\}$$.

The left-hand-side of Fig. 3 presents the graph for the static dependency relation of the foo procedure of Fig. 2. The nodes in the graph are (indexed) variables and there is an arrow from $v_1$ to $v_2$ iff $(v_1, v_2) \in SD_{\varphi _{}}$.

#### Definition 4

**(Dynamic Dependency).** The dynamic dependency relation of a program formula $\varphi _{}$ and a model ${\mu }$ of ${\varphi _{}}$ is $DD_{{\varphi _{}},{\mu }}\subseteq vars({\varphi _{}{}})\times vars({\varphi _{}{}})$ s.t.

$$DD_{{\varphi _{}},{\mu }}{}=\{(v,v_1)\ |\ \exists b,v_2\ s{.}t.\ (v=ite(b,v_1,v_2))\in S_{phi},\ {\mu }[{b}]=true\}$$

$$\cup \{(v,v_2)\ |\ \exists b,v_1\ s{.}t.\ (v=ite(b,v_1,v_2))\in S_{phi},\ {\mu }[{b}]=false\}$$

$$\cup \{(v,b)\ |\ \exists v_1,v_2\ s{.}t.\ (v=ite(b,v_1,v_2))\in S_{phi}\}$$

$$\cup \{(v,v_1)\ |\ \exists e\ s{.}t.\ (v=e)\in S_{assign},v_1\in vars({e})\}$$

Note that, dynamic dependency includes only dependencies that coincide with the specific model $\mu$, which determines whether the then or the else direction of the if is executed. Static dependency, on the other hand, takes both options into account. Thus, $DD_{{\varphi _{}},{\mu }}\subseteq SD_{\varphi _{}}$ for every model $\mu$.

The bold arrows on the right-hand-side of Fig. 3 represent the relation $DD_{{\varphi _{}},{\mu }}{}$ of the foo procedure, for any $\mu$ where ${\mu }[{g_0}]=false$.

#### Definition 5

**(Influencing Variables).** Given a program formula ${\varphi _{}}$, a model ${\mu }$ of it, and a computed variable ${v}$, the set of influencing variables of ${v}$ with respect to ${\varphi _{}}$ and ${\mu }$ is:

$${InfluenceVars_{{\varphi _{}},{\mu }}({v})}=\{v'\ |\ (v, v')\in ({DD_{{\varphi _{}},{\mu }}{}})^*\}$$

The circled nodes on the right-hand-side of Fig. 3 represents the variables that belong to ${InfluenceVars_{{\varphi _{}},{\mu }}({y_2})}$.

#### Definition 6 (Program Formula Slice)

**.** Given a program formula ${\varphi _{}}$, a model ${\mu }$ of it, and a computed variable ${v}$, the program formula slice of ${v}$ with respect to ${\varphi _{}}$ and ${\mu }$ is:

$${Slice_{{\varphi _{}},{\mu }}({v})}=\{Assign(v')\ |\ v'\in (InfluenceVars_{{\varphi _{}},{\mu }}({v})\cap ComputedVars)\}$$

Thus, intuitively, ${Slice_{{\varphi _{}},{\mu }}({v})}$ includes all constraints (in SSA form) encoding assignments that influence the value of $v$ in $\mu$. More precisely, when considering the conjunction of only the constraints of ${Slice_{{\varphi _{}},{\mu }}({v})}$, as long as the value of all input variables remains the same as in $\mu$, the value of $v$ will remain the same as well. This is formalized in the following theorem, whose proof can be found in the full version [39].

#### Theorem 2

For every $\varphi _{},\mu$ and $v$, the following holds:

$$\left[ \bigwedge _{c\in {Slice_{{\varphi _{}},{\mu }}({v})}}c \wedge \bigwedge _{v_i\in {InputVars}}(v_i={\mu }[{v_i}]) \right] \implies (v={\mu }[{v}])$$

Continuing with our example of foo procedure,

$${Slice_{{\varphi _{}},{\mu }}({y_2})}=\{ \ y_2 = ite(g_0,y_1,y_0),\ y_0 = x_0 - 3,\ g_0 = w_0 > 3 \}.$$

### Computing the Program Formula Slice

The computation of the program formula slice is composed of two steps. In the first step, we build a graph based on the static dependency relation, $SD_{\varphi _{}}$. In the second step, we compute the slice $Slice_{{\varphi _{}},{\mu }}({v})$ by computing the set of nodes reachable from ${v}$ in this graph, using a customized reachability algorithm, which makes use of the model ${\mu }$.

The graph built during the first step is called the *Static Dependency Graph (SDG)* of $\varphi _{}$. Nodes of this graph are variables of $\varphi _{}$ and edges are the static dependencies of $SD_{\varphi _{}}$. Edges are annotated using the function $\psi$, mapping every static dependency $(v,v')$ to a boolean formula such that $(v, v') \in DD_{{\varphi _{}},{\mu }}$ iff $\mu \models \psi [(v,v')]$. Specifically, for every constraint of the form $(v=ite(b,v_1,v_2))$ in $S_{phi}$, the edge $(v,v_1)$ is annotated with $b$ and the edge $(v,v_2)$ is annotated with $\lnot b$. All other edges of the graph are annotated with *true*. See the left-hand-side of Fig. 3. For simplicity all *true* annotations are omitted.

The algorithm for the second step is presented in Algorithm 1. This algorithm gets a program formula ${\varphi _{}}$, its SDG, a model $\mu$ of ${\varphi _{}}$, and a variable $v$, and computes ${Slice_{{{\varphi _{}}},{\mu }}({v})}$. First, the set ${InfluenceVars_{{\varphi _{}},{\mu }}({v})}$ is computed as the set of nodes reachable from ${v}$ in SDG, except that the reachability algorithm traverses an edge $(v,v')$ only if $\mu \models \psi [(v,v')]$. Thus, an edge $(v,v')$ is traversed iff $(v, v') \in DD_{{\varphi _{}},{\mu }}$, which means that the set of reachable nodes computed this way is in fact ${InfluenceVars_{{\varphi _{}},{\mu }}({v})}$. Finally, the slice ${Slice_{{{\varphi _{}}},{\mu }}({v})}$ is the set of constraints encoding assignments to variables in ${InfluenceVars_{{\varphi _{}},{\mu }}({v})}$.
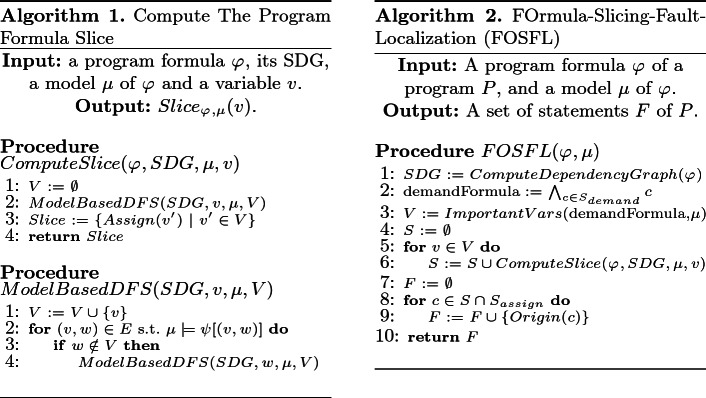


### The Fault Localization Algorithm

Our fault localization algorithm is presented in Algorithm 2. The input to this algorithm is a program formula $\varphi _{}$ of a program $P$, and a model $\mu$ of $\varphi _{}$. The model $\mu$ represents a buggy execution of $P$ on an input $I$, and the algorithm returns a set of locations, $F$, that is an $\mathcal {S}_{mut}$-must location set.

As before, we assume to know the origin of constraints in $\varphi _{}$, and use the sets $S_{assign},S_{phi}$ and $S_{demand}$. Furthermore, here we also assume that for every constraint $c\in S_{assign}$, we know exactly which program statement it came from. We call this statement the *origin* of *c*, and denote it by $Origin({c})$.

As a first step, the algorithm computes a set of variables $V$ by calling the procedure ${ImportantVars}$. This procedure receives an SMT formula $\varphi$ and a model $\mu$ of $\varphi$, and reduces $\mu$ to a partial model of $\varphi$. A *partial model* of $\varphi$ w.r.t. $\mu$ is a partial mapping from variables of the formula to values, which is consistent with $\mu$ and is sufficient to satisfy the formula. For example, for the formula $\varphi = (a=0 \vee b=0)$ and the model $\mu = \{a\mapsto 0,b\mapsto 1\}$, the valuation $\{a\mapsto 0\}$ is a partial model of $\varphi$. Procedure ${ImportantVars}$  will return the set of variables that appear in the partial model ($\{a\}$ in our example). Details of this procedure are presented in the full version [39].

The formula passed to ${ImportantVars}$ in our case is the conjunction of all demands in $S_{demand}$. Recall that the set $S_{demand}$ contains constraints encoding all conditions that need to be met for an assertion violation to happen: Conditions from assumptions appear as is, while conditions from assertions are negated and disjuncted (See Fig. 2. The last constraint on the right-hand-side represents the disjunction of the negated assertions). Therefore, the set of variables ${V}$, returned by ${ImportantVars}$, is such that as long as their values in $\mu$ remain the same, this conjunction will still be satisfied, which means that an assertion violation will still occur.

To make sure that their values do *not* remain the same, we use slicing: The algorithm proceeds by computing the program formula slice for each of the variables in ${V}$ using Algorithm 1. All slices are united into the combined set *S*. This set represents all constraints that if remain the same, then *all* the variables in ${V}$ maintain their value. Thus, at least one element from *S* must be included in any repair.

Note that, by first applying ${ImportantVars}$, we reduce the number of variables whose value should be preserved in order to maintain the bug. The smaller this number, the smaller $F$ is. We will explain the usefulness of a small $F$ in Sect. 6.

Finally, we need to translate the constraints in *S* back to statements of $P$. Because of how the slicing algorithm works, constraints in *S* may belong to either $S_{assign}$ or $S_{phi}$. If they belong to $S_{phi}$, we ignore them, because they encode the control-flow structure of the program, rather than a particular statement. Otherwise, we add the origin of the constraint, which is a statement of the program, to the set of returned locations, $F$. Note that, several different constraints may have the same origin, for example due to loop unwinding. In such a case, it is sufficient for one constraint encoding the statement $st$ to be included in *S*, for $st$ to be included in $F$. A proof for the following theorem can be found in the full version [39].

#### Theorem 3

Algorithm FOSFL is an $\mathcal {S}_{arb}$-must and also an $\mathcal {S}_{mut}$-must fault localization algorithm.

### Incremental Fault Localization

It is often necessary to apply fault localization to several bugs in the same program, or even to several programs with different bugs. Therefore, it is desired that the fault localization algorithm be *incremental*, which means that the computation effort of each fault localization attempt should be proportional to the changes made from the previous attempt. In other words, we should avoid re-computation whenever possible, taking advantage of the fact that the program remains the same, or at least remains similar.

Algorithm FOSFL can be easily made incremental for the case of different bugs of the same program. In this case, several successive calls are made to the algorithm using the same program formula $\varphi _{}$, but with different models of it. Since the static dependency relation $SD_{\varphi _{}}$ depends solely on the program formula, and not on the model, we can avoid re-computing the SDG for each call. Instead, we can compute the SDG once, upfront, and whenever FOSFL is called, simply skip the first line. We call the incremental version of FOSFL Incremental-Formula-Slicing-Fault-Localization (I-FOSFL).

Note that I-FOSFL is useful not only for fault localization of different bugs of the same program, but also whenever the SDG remains the same during successive fault localization calls. This is the case when considering different mutated programs $P'$ of the same program $P$, since every change to $P'$ replaces an expression *e* with an expression $e'$ over the same variables. Thus, the SDG remains the same, since the static dependency relation, in fact, only depends on $vars({e})$, and not on *e* itself^6^.

## Program Repair with Iterative Fault Localization

In [38], a mutation-based algorithm for program repair, named AllRepair, was presented. This algorithm uses the mutation scheme in order to repair programs with respect to assertions in the code. Unlike fault localization, where the motivation is repairing a bug for a specific input, program repair aims at repairing the program for *all* inputs. To avoid confusion, we refer to a repair for all inputs as a *full repair*. In [38], the notion of a *full repair* is bounded: loops are unwound $wb$ times, and a program is considered *fully repaired* if no assertion is violated along executions with at most $wb$ unwindings. A program that is not fully repaired is said to be *buggy*. For the rest of this section, we refer to an $\mathcal {S}_{mut}$-patch as a patch, and to an $\mathcal {S}_{mut}$-patched program as a mutated program.

As its name implies, the goal of AllRepair is to obtain all *minimal* fully repaired mutated programs, where minimality refers to the patch used in the program. It goes through an iterative generate-validate process. The generate phase chooses a mutated program from the search space, and the validate phase checks whether this program is fully repaired, by solving its program formula. The mutated program is fully repaired iff the formula is unsatisfiable.

The generate-validate process is realized using an interplay between a SAT solver and an SMT solver. The SAT solver is used for the generate stage. For every mutation *M* and line *l*, there is a boolean variable $B_M(l)$, which is true if and only if mutation *M* is applied to line *l*. A boolean formula is constructed and sent to the SAT solver, where each satisfying assignment corresponds to a program in the search space. The SMT solver is used for the validate stage. The program formula of the mutated program is solved to check if it is buggy or not. To achieve minimality, when a mutated program created using a patch $\tau$ is fully repaired, every mutated program created using a patch $\tau '$, with $\tau \subseteq \tau '$, is blocked.

**Fig. 4.**
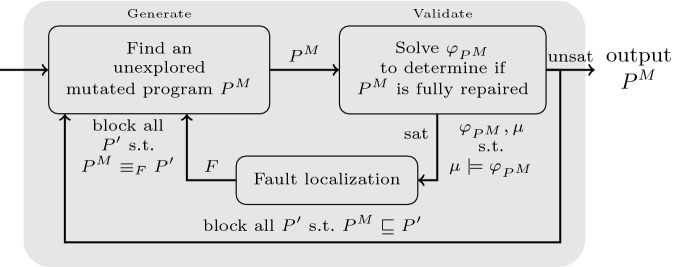
Algorithm fl-AllRepair: Mutation-based program repair with iterative fault localization. The notation ${{P^M}\equiv _{F}{P'}}$ means that $P^M$ and $P'$ agree on the content of all locations in $F$. The notation ${P^M}\sqsubseteq {P'}$ means that the patch used for creating ${P'}$ is a superset of the patch used for creating $P^M$.

### Example 4

Let $P^M$ be a fully repaired mutated program obtained by applying the patch $\tau$, consisting of mutating line $l_1$ using mutation $M_1$ and mutating line $l_2$ using mutation $M_2$. Then blocking any superset of $\tau$ will we done by adding to the boolean formula representing the search space, the blocking clause $\lnot (B_{M_1} (l_1) \wedge B_{M_2} (l_2))$, which means “either do not apply $M_1$ to $l_1$ or do not apply $M_2$ to $l_2$”. This clause blocks any mutated program with $\tau \subseteq \tau '$.

Blocking such programs prunes the search space, but only in a limited way. No pruning occurs when the mutated program is buggy.

In this paper, we extend the algorithm of [38] with a fault localization component. The goal of the new component is to prune the search space by identifying sets of mutated programs that are buggy, without inspecting each of the individual programs in the set.

Figure 4 shows the program repair algorithm with the addition of fault localization. In the new algorithm, called fl-AllRepair, whenever a mutated program is found to be buggy during the validation step, its program formula is passed to the fault localization component along with the model obtained when solving the formula. The fault localization component returns a set of locations $F$, following the I-FOSFL algorithm. Since this set is guaranteed to be an $\mathcal {S}_{mut}$-must location set, at least one of the locations in it should be changed for the bug to be fixed. Consequently, all mutated programs in which all locations from $F$ remain unchanged are blocked from being explored in the future. As before, blocking is done by adding a blocking clause that disallows such programs.

### Example 5

Let $P^M$ be a buggy mutated program for which $F$ consists of $\{ l_1, l_2, l_3 \}$, where $l_1$ was mutated with $M_1$, $l_2$ was not mutated, and $l_3$ was mutated with $M_3$. The blocking clause $\lnot B_{M_1}(l_1) \vee \lnot B_{Original}(l_2) \vee \lnot B_{M_3}(l_3)$ will be added to the boolean formula representing the search space of mutated programs. It restricts the search space to those mutated programs that either do not apply mutation $M_1$ to $l_1$, or do mutate $l_2$ or do not apply $M_3$ to $l_3$. This will prune from the search space all mutated programs which are identical to $P^M$ on the locations in $F$. Note that smaller $F$ will result in a larger set of pruned programs.

### Proposition 1

Algorithm fl-AllRepair is sound and complete.

## Experimental Results

We have implemented our fault localization technique and its integration with mutated-based program repair in the tool AllRepair, available at https://github.com/batchenRothenberg/AllRepair. In this section, we present experiments evaluating the contribution of the new fault localization component to the program repair algorithm. We refer to the algorithm of [38], without fault localization, as AllRepair, and to the algorithm presented in this paper as FL-AllRepair. Both algorithms search for minimal $wb$-violation free programs, and both are sound and complete. Thus, for every buggy program and every bound $wb$, both algorithms will eventually produce the same list of repairs.

The difference between the algorithms lies in the repair loop. In case a mutated program is found to be buggy, the AllRepair algorithm will only block the one program, while the FL-AllRepair algorithm might block a set of programs. Therefore, the number of repair iterations required to cover the search space can only decrease using the FL-AllRepair algorithm. On the other hand, the cost of each iteration with fault localization is strictly higher than without it. Our goal in this evaluation is to check if the use of fault localization pays off. That is, to check if repairs are produced faster using FL-AllRepair than using AllRepair.

*Benchmarks.* For our evaluation, we have used programs from two benchmarks: TCAS and Codeflaws. The TCAS benchmark is part of the Siemens suite [12], and is frequently used for program repair evaluation [5, 34, 38]. The TCAS program implements a traffic collision avoidance system for aircrafts, and consists of approximately 180 lines of code. We have used all 41 faulty versions of the benchmark in our experiments.

The Codeflaws benchmark [41] is also a well-known and widely used benchmark for program repair. Programs in this benchmark are taken from buggy user submissions to the programming contest site Codeforces^7^. In each program, a user tries to solve a programming problem published as part of a contest on the site. The programming problems are varied, and also the users have a diverse level of expertise. The benchmark also provides correct versions for all buggy versions, which are used to classify bug types by computing the syntactic difference. For our experiments we randomly chose 13 buggy versions classified with bug types that can be fixed using mutations. The size of the chosen programs ranges from 17 to 44 lines of code.

*Mutations.* The mutations used in AllRepair (and accordingly in FL-AllRepair) is a subset of the mutations used in [37]. We define two *mutation levels*, where level 1 contains only a subset of the mutations available in level 2. Thus, level 1 involves easier computation but may fail more often in finding repairs.

**Table 1.** Partition of mutations to levels

Level 1	Level 2
{$+,-$}, {$/,\%$}	{$+,-,*$}, {$/,\%$}
$\{>,>=\}$, $\{<,<=\}$	$\{>,>=,<,<=\}$, $\{==,!=\}$
{||, & & }	
{$>>, <<$},{&, |, ⌃}	
	C $\rightarrow$ C +1, C $\rightarrow$ C −1, C $\rightarrow$ −C, C $\rightarrow$ 0

Table 1 shows the list of mutations used in each mutation level. For example, for the category of arithmetic operator replacement, in mutation level 1, the table specifies two sets: $\{+,-\}$ and $\{/,\%\}$. This means that a + can be replaced by a − , and vice versa, and that the operators /, % can be replaced with each other. Constant manipulation mutations apply to a numeric constant and include increasing its value by 1 (C $\rightarrow$ C +1), decreasing it by 1 (C $\rightarrow$ C −1), setting it to 0 (C $\rightarrow$ 0) and changing its sign (C $\rightarrow$ −C).

*Setting.* All of our experiments were run on a Linux 64-bit Ubuntu 16.0.4 virtual machine with 1 CPU, 4 GB of RAM and 40 GB of storage, provided using the Vmware vRA service^8^. For each of the buggy versions in our benchmarks we have experimented with both mutation levels 1 and 2. For the Codeflaws benchmarks we additionally experimented with different unwinding bounds: 2 (entering the loop once), 5, 8 and 10. This experiment is irrelevant to the TCAS benchmarks since the TCAS program does not contain loops or recursive calls. Overall we had 186 combinations of buggy programs, mutation levels and unwinding bounds. We refer to each such combination as an *input*. For each input, we run both the AllRepair and the FL-AllRepair algorithms with a timeout of 10 minutes and a mutation size limit of 2 (i.e., at most two mutations could be applied at once).

### Results

In total, 131 different repairs were found during our experiments, for 60 different inputs (for several inputs there was more than one possible repair). In this count, we treat repairs fixing the same program in the same way as different, if they were produced using different mutation levels or unwinding bounds. This is because our evaluation is concerned with the time to find these repairs, and both the mutation level and the unwinding bound greatly influence this time.

Because the time to produce a repair sometimes varied in several orders of magnitude depending on the input, we have chosen to split repairs into three categories: fast, intermediate, and slow, and examine the time difference separately for each category. Splitting repairs to categories was done according to the time it took to find them using the AllRepair algorithm. If that time was under 5 seconds, the repair was considered fast. If it was over 4 minutes, it was considered slow, and otherwise it was considered intermediate.

**Fig. 5.**
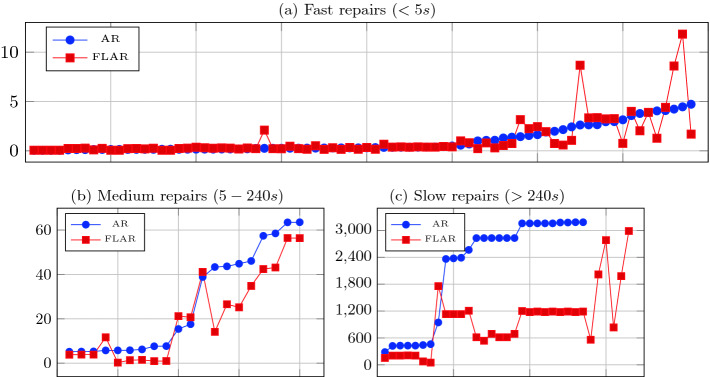
Time to find each repair using AllRepair (AR) and FL-AllRepair (FLAR). Each x value represents a single repair, and the corresponding y values represent the time, in seconds, it took to find that repair using both algorithms. Note that the graphs differ in the y axis scale.

Figure 5 shows a comparison of the time, in seconds, it took to find repairs in both algorithms. There are three graphs, according to our three categories. In all graphs, each x value represents a single repair, where the corresponding blue dot in the y axis represents the time it took to find that repair using AllRepair, and the red square represents the time using FL-AllRepair. So, whenever the blue dot is above the red square, FL-AllRepair was faster in finding that repair, and the y difference represents the time saved.

For the fast category (Fig. 5a), there is no clear advantage to FL-AllRepair. The majority of the repairs in this category are produced in less than a second using both algorithms. For the remaining repairs, there appears to be as many cases where FL-AllRepair is faster as when it is slower. But, in all cases where there is a time difference, in either direction, it is only of a few seconds.

For the intermediate category (Fig. 5b), the advantage of FL-AllRepair is starting to become clear. There are now only 4 repairs (out of 20) for which FL-AllRepair is slower. Also, on average, it is slower by 4 seconds, but faster by 10 seconds. Finally, for the slow category (Fig. 5c), there is an obvious advantage to FL-AllRepair. First, it is able to find 6 repairs *exclusively*, while AllRepair reaches a time-out. Also, for the remaining 27 repairs, FL-AllRepair is faster in all cases but one. The time difference is now also very significant: FL-AllRepair is faster by 1512 seconds (around 25 minutes) on average.

To sum up, the results show that in many cases our algorithm FL-AllRepair is able to save time in finding repairs. The savings are especially significant in cases where it takes a long time to produce the repair using the original AllRepair algorithm, and these are the cases where time savings are most needed.

### Comparison with Other Repair Methods

The TCAS benchmark was recently used also in [34], where AllRepair’s performance was compared to that of four other automated repair tools: Angelix [29], GenProg [26], FoRenSiC [5] and Maple [34]. AllRepair was found to be faster by an order of magnitude than all of the compared tools, taking only 16.9  seconds to find a repair on average, where the other tools take 1540.7, 325.4, 360.1, and 155.3 seconds, respectively. Since in our experiments on TCAS fl-AllRepair was faster than AllRepair on average (and even when it was slower it was only by a few seconds), we conclude that fl-AllRepair also compares favorably to these other tools.

In terms of repairability, the repair scheme used by AllRepair (and fl-AllRepair) is limited compared to the other tools: AllRepair only uses mutations on expressions while Angelix, FoRenSiC and Maple allow replacing an expression with a template (e.g., a linear combination of variables), which is then filled out to create a repair. GenProg allows modifying a statement as well as deleting it or adding a statement after it. Therefore, the other tools are inherently capable of producing repairs in more cases than AllRepair.

In the case of TCAS, the study showed that AllRepair is able to find repairs for 18 versions (a result that we confirm in our experiments as well), while Angelix, GenProg, FoRenSiC and Maple found 32, 11, 23 and 26, respectively. But, what the study also showed, is that in repair methods that are based on tests, in many cases the repair found only adhered to the test-suite, but was not correct when inspected manually. When counting only correct repairs, AllRepair finds repairs for 18 versions (all of AllRepairs repairs are correct), while Angelix, GenProg, FoRenSiC and Maple find 9, 0, 15 and 26, respectively. Since fl-AllRepair is able to find all repairs found by AllRepair, the same results also apply to fl-AllRepair.

## Related Work

Dynamic slicing has been widely used for fault localization in the past [16, 36, 43, 45–47]. But, as we have seen, traditional notations of dynamic slicing [2, 23] are not must (with respect to neither of the presented schemes), and thus, the above techniques may fail to include relevant locations in their results.

Other approaches for fault localization include spectrum-based (SBFL) [1, 13, 20, 31, 44], mutation-based (MBFL) [15, 18, 30, 35] and formula-based (FBFL) [7, 14, 17, 21, 40]. Both SBFL and MBFL techniques compute the suspiciousness of a statement using coverage information from failing and passing test executions. MBFL uses, in addition, information on how test results change after applying different mutations to the program. Both SBFL and MBFL techniques can be seen as may fault localization techniques, in nature: they return locations that *are likely* to be relevant to the failing execution, based on all executions. We see may fault localization techniques as orthogonal to ours (and to must fault localization techniques in general), since in the trade-off between returning a small set of locations, and returning one that is guaranteed to contain all relevant statements, may techniques prefer the first, while must techniques prefer the second. In the context of repair, there are interesting applications for both.

FBFL techniques represent an error trace using an SMT formula and analyze it to find suspicious locations. These techniques include using error invariants [6, 14, 17, 40], maximum satisfiability [21, 24, 25], and weakest preconditions [7]. What we were able to show in this paper, is that the methods of [6, 14, 21] are not must. In contrast, we believe (though we do not prove it) that the methods of [7, 24, 25] are must. But, what [7, 24, 25] have in common is that they use the semantics of the error trace or the program. Though semantic information can help to further minimize the number of suspicious locations, retrieving it involves using expensive solving-based procedures. Our approach, on the other hand, uses only syntactic information, which makes the fault localization computation relatively cheap; No SMT solving is needed. Thus, these approaches can be seen as complementary to ours.

In the literature there is also a wide range of techniques for automated program repair using formal methods [4, 10, 19, 22, 29, 32, 33, 42]. Both [11] and [37] also use fault localization followed by applying mutations for repair. But, unlike this work, fault localization is applied only for the original program. Also, neither the Tarantula fault localization used in [11] nor the dynamic slicing used in [37] carries the guarantee of being a must fault localization. The tool MUT-APR [3] fixes binary operator faults in C programs, but only targets faults that require one line modification. The tools FoREnSiC [5] and Maple [34] repair C programs with respect to a formal specification, but they do so by replacing expressions with templates, which are then patched and analysed. SemGraft [28] conducts repair with respect to a reference implementation, but relies on tests for SBFL fault localization of the original program.

## Conclusion

In this work we define a novel notion of *must* fault localization, that carefully identifies program locations that are relevant for a bug, so that the set is sufficiently small but is guaranteed not to miss desired repairs. We also show that the notion of *must* fault localization should be defined with respect to the repair scheme in use. We show that our notion of must fault localization is particularly useful in pruning the search space of a specific mutation-based repair algorithm.

To the best of our knowledge, we are the first to investigate the widely-used notion of fault localization and to suggest criteria for evaluating its different implementation.

## Footnotes

1 Alternatively, one could assume to know the desired output of the program for $I$ and define a bug on $I$ as a case where the program outputs the wrong value for $I$.

2 We sometimes omit $\mathcal {S}$ from notations where $\mathcal {S}$ is clear from context.

3 If $st$ is an assignment, its expression is its right-hand-side. If $st$ is a conditional statement, its expression is its condition.

4 This simple definition of the mutation scheme is used only for simplicity of presentation. Our implementation supports a much richer set of mutation rules, as explained in Sect. 7.

5 This is, in fact, a hitting set of the set of all minimal $\mathcal {S}$-repairable sets.

6 This is true for $\mathcal {S}_{mut}$ but not for $\mathcal {S}_{arb}$, since the latter allows to replace an expression *e* with an expression $e'$ over different variables.

7 http://codeforces.com/.

8 https://www.vmware.com/il/products/vrealize-automation.html.
